# Prostate Tuberculosis: six forms of clinical presentation

**DOI:** 10.1590/S1677-5538.IBJU.2023.0299

**Published:** 2024-03-18

**Authors:** André Avarese Figueiredo, Humberto Elias Lopes, Augusto de Azevedo Barreto, Victor Silvestre Soares Fanni, José Murillo Bastos

**Affiliations:** 1 Universidade Federal de Juiz de Fora Núcleo Interdisciplnar de Pesquisa em Urologia MG Brasil Núcleo Interdisciplnar de Pesquisa em Urologia - NIPU, Universidade Federal de Juiz de Fora, MG, Brasil; 2 Universidade Federal de Juiz de Fora Departamento de Cirurgia MG Brasil epartamento de Cirurgia, Universidade Federal de Juiz de Fora, MG, Brasil

## INTRODUCTION

Urogenital tuberculosis (UGT) is an old disease that is still little known and often forgotten by urologists; the three main questions regarding when to suspect, how to diagnose, and when to treat UGT remain a not well-stablished topic. These problems result from the rarity of UGT, lack of disease knowledge by physicians, nonspecific symptoms, and low sensitivity of bacteriological tests (1). Prostate tuberculosis is even more rare and unknown. It is classically described as predominantly subclinical and is almost always an accidental finding in patients undergoing transurethral resection of the prostate (TURP) (2). However, some authors have identified possible variations in the clinical manifestations of prostate tuberculosis, such as chronic prostatitis (3, 4) and the formation of prostatic abscesses in patients without immunodeficiencies (4, 5). Thus, there is a need to describe the possible clinical presentation forms of prostate tuberculosis, which will have evident implications in improving clinical suspicion and diagnosis.

Quantitative studies analyze data using statistical calculations to characterize the best diagnostic test and the associations between causes and effects and between interventions and results. Quantitative studies focus on the question of "How much?" and on what is the mean response of a specific population. However, certain questions and scientific hypotheses cannot be answered by this type of study. Qualitative studies evaluate individual responses and focus on the "How?" and "Why?" (6). As a goal of this study, we have the following questions: How does prostate tuberculosis manifest clinically and how the diagnosis is made?

There are currently no clear answers to these questions. Thus, the objective of the present study was to evaluate a series of patients diagnosed with prostate tuberculosis using qualitative methodology to define through codification the clinical presentations and characterize the diagnostic roadmap of the disease.

### Patients and methods

A retrospective qualitative study of hospital records of patients diagnosed with prostate tuberculosis was performed. Content analysis was used as the theoretical basis of the study, which is characterized by the systematic organization of information in a code structure (7).

Patients selected for the study included 18 men diagnosed with prostate tuberculosis in two public tertiary hospitals. Patients were selected by convenience after identification of the diagnosis of prostate tuberculosis in the hospital archives.

Patients were male, aged between 26 and 75 years, and diagnosed with prostate tuberculosis by bacteriology (six patients), histological examination (nine patients), and therapeutic tests (three patients). The patients diagnosed by therapeutic testing, despite a lack of bacteriological and histological confirmation, underwent specific treatment for tuberculosis due to high clinical and radiological suspicion and presented resolution of symptoms.

To characterize prostate tuberculosis presentation, the hospital records and laboratory, radiological, and histopathological examinations were analyzed for each patient. The following information were collected: age, comorbidities, signs and symptoms, method of diagnosis of tuberculosis, presence of concomitant urinary tuberculosis or extra urinary tuberculosis, and disease progression. After data collection, inductive analysis was performed, and a code list was created with the title "Clinical presentation". A code was created to identify each form of clinical presentation or occurrence of associated urinary tuberculosis.

Patient information was used to describe the "Diagnostic Roadmap", which characterized each patient based on a) the type of clinical presentation; b) the presence or absence of suspected prostate tuberculosis by the attending physician; c) information on test results relevant to the diagnosis; d) diagnostic criteria for prostate tuberculosis; and e) results of the histopathological analysis, when performed.

After the codes were analyzed, clinical presentations of prostate tuberculosis and clinical, laboratory, radiological, and histological characteristics of the disease were described, and an illustrative map of the pathophysiology was created.

Eleven codes were created from the information collected from the 18 patients with prostate tuberculosis. The codes and code description are:

1. **Asymptomatic**
  Patient with an incidental histological diagnosis of prostate tuberculosis on autopsy after radical prostatectomy for treatment of prostate cancer or prostate biopsy in the absence of the following symptoms: dysuria, pelvic pain, genital pain, perineal pain, and lower urinary tract symptoms (LUTS).
2. **Renal cortical tuberculosis**
  Patient with prostate tuberculosis associated with bilateral renal cortical tuberculosis, without excretory involvement, characterized by synchronous involvement of the renal parenchyma and prostate.
3. **Without urinary tuberculosis**
  Patient with prostate tuberculosis but with a normal urinary tract on imaging tests.
4. **Abscess**
  Patient with prostate tuberculosis and clinical and radiological presentation with abscess formation in the prostate and with spontaneous drainage into the perineum or rectum or requiring rectal, perineal, or transurethral drainage.
5. **Chronic epididymitis**
  Patient with prostate tuberculosis associated with unilateral or bilateral epididymitis, with clinical presentation in the form of pain and increased epididymal volume/nodulation, or epididymal abscess with or without epididymal cutaneous fistula, lasting more than three months.
6. **Contracted bladder**
  Patient with prostate tuberculosis associated with bladder contracted by tuberculosis, which means a low compliance bladder with less than 100 mL of capacity.
7. **Chronic prostatitis**
  Patient with prostate tuberculosis with clinical presentation in the form of chronic prostatitis characterized by recurrent or persistent symptoms for more than three months including pain (perineal, pelvic, or genital) or dysuria associated or not with LUTS.
8. **Obstruction**
  Patient with prostate tuberculosis with clinical presentation of obstructive LUTS with necessity of surgery.
9. **Unilateral renal tuberculosis**
  Patient with prostate tuberculosis associated with unilateral renal tuberculosis with stenosis and obstruction of the excretory tracts and functional exclusion of the kidney.
10. **Acute prostatitis**
  Patient with prostate tuberculosis with clinical presentation of recurrent acute prostatitis (more or equal to three episodes per year) with fever, dysuria, perineal pain, and LUTS at onset or recent worsening, with clinical improvement after conventional antibiotic therapy.
11. **BCG**
  Patient with prostate tuberculosis with a previous history of intravesical BCG instillation for the treatment of superficial bladder carcinoma.

## RESULTS

### Clinical Presentation

In the code analysis, we found that prostate tuberculosis can present clinically in six forms:

1. **Asymptomatic:**
  Four patients were asymptomatic for prostatic symptoms. One patient was diagnosed after autopsy and finding of tuberculosis foci in the prostate and in bilateral renal parenchyma. Two patients were diagnosed after prostate biopsy by suspicion of prostate cancer. The fourth patient who was diagnosed with prostate cancer and chronic pain in the left epididymis underwent radical prostatectomy and left orchiectomy surgery and had a histological diagnosis of prostate cancer associated with tuberculosis in the prostate, left seminal vesicle, and left epididymis.
2. **Prostate obstruction and LUTS:**
  Seven patients had LUTS secondary to prostatic obstruction requiring TURP, which was performed in all patients but one still in use of an indwelling urinary catheter.
3. **Chronic prostatitis:**
  Eight patients presented with chronic prostatitis, classified according to the NIH index as chronic prostatitis type III (8).
4. Recurring acute prostatitis:
  Two patients had recurrent episodes of acute prostatitis (three or more per year) with temporary resolution of symptoms with the use of conventional antibiotic therapy. In these patients, urine culture during the acute infection was negative or there was growth of the usual bacteria (Escherichia coli).
5. **Prostate abscess:**
  Two patients presented with a prostate abscess requiring rectal or urethral drainage.
6. **Chronic epididymitis:**
  Seven patients presented with chronic epididymitis with pain and tumor in the epididymis, which was unilateral (six cases) or bilateral (one case) and with a cutaneous epididymal fistula in one case. The presence of epididymitis was associated with all other clinical presentation forms and was the only clinical manifestation of prostate tuberculosis in an asymptomatic patient, as previously described. Two patients who underwent evaluation of the seminal vesicles (histologically after radical prostatectomy and radiologically after nuclear magnetic resonance) showed extensive involvement of the seminal vesicle ipsilateral to the epididymitis.

**Figure 1:**
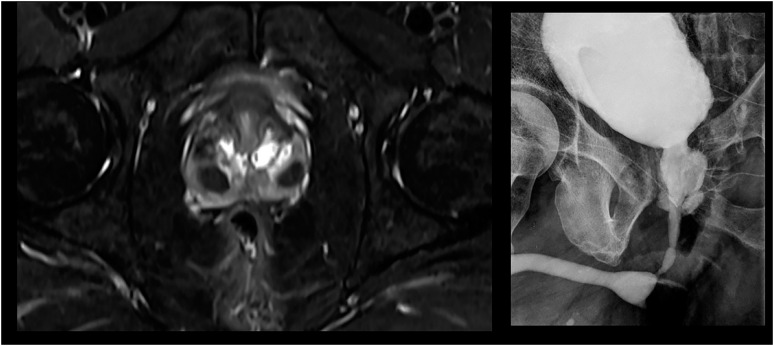
Pathophysiology of prostate tuberculosis six forms of presentation.

By analyzing the six clinical presentation forms, we characterized three types of prostate tuberculosis progression after activation of the disease (Figure-1):

**Prostate tuberculosis occurs after hematogenous dissemination from pulmonary tuberculosis or through urinary dissemination from the bladder bacillus. After activation, the prostate may present: a) morphological changes with nodule formation or diffuse enlargement causing prostatic obstruction or cancer suspicion in asymptomatic cases; b) prostate infection/ inflammation causing recurring acute prostatitis, chronic prostatitis or abscess formation; and c) canalicular dissemination to the epididymis and may cause chronic epididymitis.**

**Figure 2:**
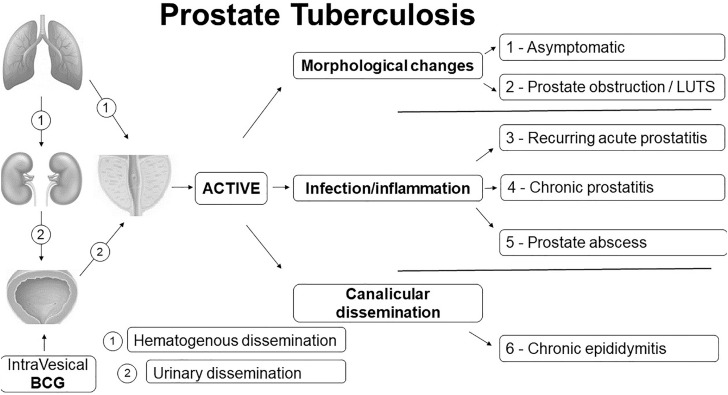
Pelvic magnetic resonance.

1. Morphological changes: appearance of nodules and enlarged prostate with eventual urinary obstruction. In fact, present cases prostate images have shown mostly diffuse lesions without nodular formation, but also single or multiple nodular or cystic lesions and a more specific finding with destruction of prostatic parenchyma called auto-prostatectomy (Figure-2).
2. Infection/inflammation: appearance of infectious symptoms with pain and eventual formation of prostatic abscess.
3. Canalicular dissemination: appearance of epididymitis secondary to prostatic disease.

**(A) Left image: Pelvic magnetic resonance of prostate tuberculosis in a 34-year-old male with chronic prostatitis: axial T2-weighted image shows bilateral low signal intensity nodules in peripheric prostatic zone and multiple cystic lesions in transition zone. (B) Right image: Retrograde urethrocystography of a 60-year-old man with recurring acute prostatitis shows an autoprostatectomy pattern of prostate tuberculosis with widely dilated posterior urethra with prostatic cavitation, due to prostatic parenchyma destruction.**

Prostate tuberculosis was associated with urinary tuberculosis in nine patients, as follows: a) bilateral parenchymal renal tuberculosis at autopsy (one case), b) unilateral renal tuberculosis (three cases), and c) bladder tuberculosis with contracted bladder (five cases). In nine patients, prostate tuberculosis was not associated with changes in the urinary tract, either clinically or radiologically. Of these cases, in two patients, prostate tuberculosis was secondary to intravesical Bacillus Calmette-Guérin (BCG) application to prevent recurrence of noninvasive bladder cancer.

### Diagnostic Roadmap

In nine patients, there was no suspicion of prostate tuberculosis, and the diagnosis was made accidentally by histology after a) TURP of patients with prostatic obstruction (four cases); b) prostate biopsy for suspected prostate cancer (two cases); c) radical prostatectomy (one case); d) after necropsy in an asymptomatic patient (one case); and e) after exclusion kidney nephrectomy in a patient with chronic prostatitis (one case).

In nine patients, prostate tuberculosis was suspected due to the presence of a) chronic prostatitis (six cases); b) acute recurrent prostatitis (two cases); and c) prostatic abscess (one case). In these patients, upon suspicion, an acid-alcohol resistant bacillus culture of the urine and drained abscess was requested. The culture was positive in seven cases and negative in three cases (70% sensitivity). Patients with chronic prostatitis and negative culture results were subjected to a serum Interferon-Gamma Release Assay (IGRA), which was positive in one patient and negative in two patients (33.3% sensitivity). In these three cases without bacteriological or histological confirmation of tuberculosis, a successful therapeutic test was performed, with resolution of chronic prostatitis symptoms after treatment.

Eleven patients had prostate histological examination data from autopsy (one case), TURP (seven cases), prostate biopsy (two cases), or a radical prostatectomy specimen (one case). Granulomatous prostatitis was found in eight cases (seven with caseous necrosis and one case after BCG without caseous necrosis, only with epithelioid granulomas) i.e., in 72.7 % of cases. In three cases the histological result was benign prostatic hyperplasia.

## DISCUSSION

One of our asymptomatic patients had tuberculosis in the prostate and in bilateral renal parenchyma. Approximately two of three patients who die from tuberculosis of any origin have tuberculosis in the prostate (3). In a study of 27 patients who received intravesical BCG and were subsequently subjected to cystoprostatectomy, granulomatous prostatitis was found in 81.5% of cases (9). Despite the absence of symptoms in these patients, there was a predominance of granulomas with caseous necrosis up to 3 cm in size. Therefore, the prevalence of tuberculosis in the prostate, either after BCG or after clinical tuberculosis, is high, contrasting with the low prevalence of clinical symptoms. The significance of this latent prostatic tuberculosis is still unknown. It is unknown whether or when progression to the symptomatic form of the disease will occur. In review articles on UGT, prostate tuberculosis is described as having subclinical evolution, with an incidental finding in patients undergoing surgical treatment of benign prostatic hyperplasia or after prostate biopsy for suspected prostate cancer (1, 10). These two situations occurred in our patients, which suggested that during the disease evolution, morphological changes including nodule formation occur, simulating prostate cancer and gland growth, which can cause prostate cancer suspicion and prostatic obstruction.

In the present series, seven patients had prostatic obstruction requiring TURP. This is an old known association. In a 1915 publication, Koll et al. described the case of a 61-year-old patient with urinary retention and histological diagnosis after prostatectomy of extensive tuberculosis in the prostate (11) In 1951, Barker et al. described five patients with prostate tuberculosis with LUTS and two patients with prostatic obstruction with prostate nodules but without other abnormalities (12). Importantly, a 55-year-old patient with LUTS and prostatic nodules had a histological diagnosis of prostate tuberculosis by biopsy and showed resolution of urinary symptoms after specific pharmacological treatment (13).

The progression of prostate tuberculosis through the infection/inflammation route was the most important finding, occurring in 12 of the 18 patients. Chronic prostatitis with negative urine culture occurred in eight patients and could be considered chronic pelvic pain syndrome. In the European Association of Urology guideline for chronic pelvic pain syndrome, primary prostate pain syndrome is described as "persistent or recurrent episodic pain perceived in the prostate for ≥3 months with no proven infection or obvious local pathology" (14) In the guideline, there is no reference to tuberculosis as a possible cause of chronic prostatitis, showing that this hypothesis has been neglected. In the evaluation of three recent reviews (10, 15, 16), the association between prostatic tuberculosis and chronic prostatitis or chronic pelvic pain syndrome is practically nonexistent. However, the importance of tuberculosis in the etiology of chronic prostatitis has been emphasized by some authors. In Russia, Kulchavenya et al. analyzed a cohort of 73 patients with chronic prostatitis. Tuberculosis prostatitis was diagnosed in 17 patients (23.3%) (17). In this article, as in our study, the presence of usual bacteria occurred in patients with chronic tuberculous prostatitis. The most severe evolution of infectious/inflammatory manifestations of prostate tuberculosis is the formation of abscesses. The two cases described in this article occurred in young patients (26 and 30 years) without AIDS or evident immunosuppression. Although the occurrence of prostatic abscess due to tuberculosis is associated with AIDS (16), it may occur in patients after BCG administration and without immunosuppression (18).

In male genital tuberculosis, the most common clinical presentation is chronic epididymitis (1). In two of our cases of epididymitis, the ipsilateral seminal vesicle was also affected by tuberculosis, reinforcing the hypothesis of canalicular spread of tuberculosis. There is a well-established concept that the bacillus can reach the epididymis via the retrograde sperm route but that the main route is hematogenous (16). This is explained by the clinical aspect, when in most cases epididymitis is the only genital symptom. However, in a classic autopsy study, all cases of epididymal tuberculosis were associated with prostate tuberculosis, and cases of isolated prostatic tuberculosis were described (19). All these data support the concept that in genital tuberculosis, initial prostatic involvement and subsequent scrotal canalicular dissemination often occur.

In conclusion, prostate tuberculosis is a disease of low suspicion and difficult diagnosis. Prostate tuberculosis manifests in well-defined six forms of clinical presentation: asymptomatic, prostate obstruction and LUTS, chronic prostatitis, recurring acute prostatitis, prostate abscess and chronic epididymitis.
